# Recent Advances in Metabolic Pathways of Sulfate Reduction in Intestinal Bacteria

**DOI:** 10.3390/cells9030698

**Published:** 2020-03-12

**Authors:** Ivan Kushkevych, Jiří Cejnar, Jakub Treml, Dani Dordević, Peter Kollar, Monika Vítězová

**Affiliations:** 1 Department of Experimental Biology, Faculty of Science, Masaryk University, Kamenice 753/5, 62500 Brno, Czech Republic; 451355@mail.muni.cz (J.C.); vitezova@sci.muni.cz (M.V.); 2 Department of Molecular Biology and Pharmaceutical Biotechnology, University of Veterinary and Pharmaceutical Sciences Brno, 61242 Brno, Czech Republic; tremlj@vfu.cz; 3 Department of Plant Origin Foodstuffs Hygiene and Technology, Faculty of Veterinary Hygiene and Ecology, University of Veterinary and Pharmaceutical Sciences, 61242 Brno, Czech Republic; dani_dordevic@yahoo.com; 4 Department of Human Pharmacology and Toxicology, Faculty of Pharmacy, University of Veterinary and Pharmaceutical Sciences, 61242 Brno, Czech Republic; kollarp@vfu.cz

**Keywords:** intestinal microbiota, sulfate reduction, assimilatory, sulfate-reducing bacteria, hydrogen sulfide, toxicity, cysteine biosynthesis

## Abstract

Sulfate is present in foods, beverages, and drinking water. Its reduction and concentration in the gut depend on the intestinal microbiome activity, especially sulfate-reducing bacteria (SRB), which can be involved in inflammatory bowel disease (IBD). Assimilatory sulfate reduction (ASR) is present in all living organisms. In this process, sulfate is reduced to hydrogen sulfide and then included in cysteine and methionine biosynthesis. In contrast to assimilatory sulfate reduction, the dissimilatory process is typical for SRB. A terminal product of this metabolism pathway is hydrogen sulfide, which can be involved in gut inflammation and also causes problems in industries (due to corrosion effects). The aim of the review was to compare assimilatory and dissimilatory sulfate reduction (DSR). These processes occur in some species of intestinal bacteria (e.g., *Escherichia* and *Desulfovibrio* genera). The main attention was focused on the description of genes and their location in selected strains. Their coding expression of the enzymes is associated with anabolic processes in various intestinal bacteria. These analyzed recent advances can be important factors for proposing possibilities of metabolic pathway extension from hydrogen sulfide to cysteine in intestinal SRB. The switch from the DSR metabolic pathway to the ASR metabolic pathway is important since toxic sulfide is not produced as a final product.

## 1. Introduction

Sulfur is an indispensable element for living organisms, including prokaryotes and eukaryotes. This element is important in the synthesis of amino acids, proteins, and enzymes [1]. Sulfur metabolism depends on different organisms and the environment. These factors determine its oxidation state. Microorganisms can involve sulfur in their metabolism, both in oxidized and reduced states [2].

The intestinal microorganisms utilize mostly the oxidized state of sulfur from various complex organic compounds (such as sulfate present in food and water). The intestinal microbiome has a key impact on human health and can be involved in immunity, metabolism, and neurobehavioral traits. Although intestinal bacteria have been studied for several decades, their role in the intestines has been found more interesting than classical infectious microorganisms [3].

The reduction of sulfate, depending on microorganism, can take place in two ways: assimilatory and dissimilatory (differing in the final product) [1,2]. In dissimilatory sulfate reduction (DSR), toxic hydrogen sulfide is produced, and in an assimilatory way, the terminal product is cysteine. One possible hypothesis of intestinal inflammation, including ulcerative colitis, is the effect of high concentrations of hydrogen sulfide on the intestinal epithelium [4,5,6,7,8,9,10]. In recent years, intestinal sulfate-reducing bacteria (SRB) (as a common part of the intestinal microbiome) have been associated with this disease [11,12,13].

SRB are anaerobic bacteria that use organic compounds as a source of energy and carbon in a process called DSR [14,15,16,17]. This is typical of only five bacterial strains [18]. In this metabolic pathway, sulfate is used as an electron acceptor and is reduced to hydrogen sulfide, which is subsequently released into the intestine [4,19,20,21]. SRB are not the only bacteria that use sulfate and produce hydrogen sulfide. There are some other bacteria in the intestine, such as *Escherichia coli*, that metabolize sulfate to hydrogen sulfide, and further to sulfur amino acids. This metabolic pathway is called assimilatory sulfate reduction (ASR) and occurs in both bacteria and plants [22].

The aim of the review was to compare two different metabolic pathways of sulfate in bacteria. There is information in the literature about individual genes and localization, but a comparison of gene localization and genes themselves has not yet been described. The most noticeable differences between the above pathways is that dissimilate sulfate serves to generate energy and sulfate is an electron acceptor. During assimilatory sulfate reduction, sulfate is used in the synthesis of amino acids. In these pathways, several differences (the inclusion of different enzymes that lead to distinct final products) can be observed at the enzyme and gene levels. These findings can help in finding a solution for H_2_S overproduction.

## 2. Sulfate Source for Intestinal Bacteria

The sulfate contained in food plays an important role in human metabolism. It is involved in the formation of methionine and cysteine. In conjugation with xenobiotics and drugs, sulfate influences the metabolism of the large intestine, where it is reduced by bacteria to toxic and harmful intestine hydrogen sulfide [12,13]. However, it is poorly absorbed in the intestine and can be used as a laxative or contrast agent, such as Ba_2_SO_4_. An adult human body receives more than 16 mmol of sulfate per day [23]. A portion of the sulfate is absorbed in the small intestine and is used to form sulfur-containing compounds, such as chondroitin sulfate and mucin, or is used for the synthesis of amino acids methionine and cysteine [23]. Cysteine is an indispensable component of peptides, involved in the formation of disulfide bridges, and is highly represented in keratin protein. Keratin protein can be found in hair and nails, and it is included in the formation of taurine. Cysteine decarboxylation produces mercaptoethanolamine, which is involved in the biosynthesis of coenzyme A [24]. The remaining sulfate ions that are not absorbed in the small intestine, reach the colon, where they serve as electron acceptors for SRB [4]. The largest sources of sulfate in the diet are industrially processed foods such as wheat bread (15 μmol/g), sausages (10 μmol/g), but also nuts (9 μmol/g) and dried fruit (10 μmol/g). A high sulfate content occurs in beer (2.6 μmol/mL) and some vegetables (8.4 μmol/g); mainly vegetables, the most often from the cabbage family, such as cauliflower, which is a rich source of glucosinolates. Their general formula is R-C-(NOSO_3_)-S-glucose [23]. Besides sulfate, there are other sulfur compounds in the intestine. Other intestinal bacteria are involved in the processing of these sulfur compounds (Table 1), which are necessary to maintain a balance between the harmful and beneficial effects of sulfur-containing compounds on the intestinal tract [2].

## 3. Dissimilatory Sulfate Reduction

Only a special group of organisms can extract energy from DSR, while the ASR process is abundant among other organisms (plants and bacteria). SRB are an important group of microorganisms in various ecosystems, including the intestinal tract, because they require inorganic sulfate as an electron acceptor to obtain energy from organic compounds [25,26]. SRB are a significant part of the environment because they require inorganic sulfate for energy production [18]. DSR is a process that includes several reactions, providing energy for the creation of adenosine triphosphate (ATP) in bacterial cells of SRB.

**Table 1:** Various bacterial genera involved in sulfur metabolism (modified from Carbonero et al. 2012) [2].

Source of Sulfate	Substrates Containing Sulfur	Bacteria Genera	Reference
Inorganic	Sulfate (SO_4_^2−^)	*Desulfovibrio* sp.	Gibson et al. 1988 [12]
		*Desulfobacter* sp.	
		*Desulfobulbus* sp.	
		*Desulfotomaculum* sp.	
	Sulfite (SO_3_^2−^)	*Bilophila wadsworthia*	Baron et al. 1989 [27]
		*Campylobacter jejuni*	Kelly and Myers 2005 [28]
Organic	Cysteine	*Escherichia coli*	Metaxas and Delwiche 1955 [29]
		*Staphylococcus aureus*	Shatalin et al. 2011 [30]
		*Salmonella typhimurium*	Kredich et al. 1972 [31]
		*Mycobacterium tuberculosis*	Wheeler et al. 2005 [32]
		*Helicobacter pylori*	Kim et al. 2006 [33]
		*Prevotella intermedia*	Igarashi et al. 2009 [34]
		*Fusobacterium nucleatum*	Yoshida et al. 2010 [35]
		*Streptococcus anginosus*	Yoshida et al. 2010 [36]
		*Clostridium* sp.	Genom cysteine desulfohydrase
		*Enterobacter* sp.	Genom cysteine desulfohydrase
		*Klebsiella* sp.	Genom cysteine desulfohydrase
	Sulfomucin	*Prevotella* strain RS2	Roberton et al. 2000 [37]
		*Bacteroides fragilis*	Roberton et al. 2000 [37]
		*Helicobacter pylori*	Slomiany et al. 1992 [38]
		*Akkermansia* sp.	Genom glycosulfatase

**Figure 1:**
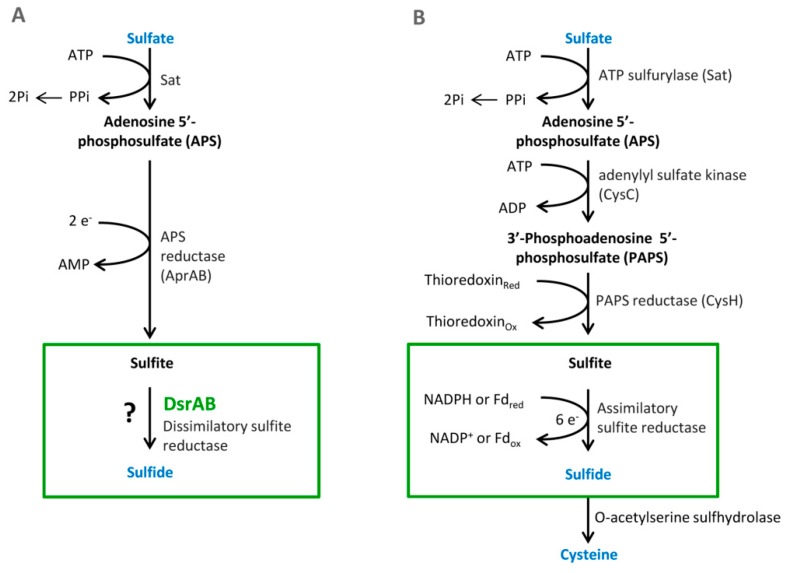
The pathways of the dissimilatory (**A**) and assimilatory (**B**) sulfate reduction, from Santos et al. 2015 [47].

Sulfate is used as a terminal acceptor and provides energy for growth and is released from the oxidation of organic compounds (lactate, propionate, and butyrate) or hydrogen [39,40]. Reduction of sulfate occurs over many different intermediates that are not released to the environment. The process of DSR includes the transfer of eight electrons [4]. Enzymes that are associated with this process are localized in the cytoplasm or periplasm of the bacterial cells. The first step is sulfate and proton absorption into bacterial cells [41,42,43,44,45]. After these processes, DSR can be divided into six stages (Figure 1).

### 3.1. Sulfate Activation

Sulfate must be reduced and activated before absorption. This enzymatic reaction is catalyzed by the enzyme ATP sulfurylase (EC 2.7.7.4.). The products of this reaction are adenosine-5-phosphosulfate (APS) and pyrophosphate (PP_i_) [14]. Firstly, free sulfate around bacterial cells must be activated by APS sulfurylase that binds ATP to sulfate. This enzyme is part of three metabolic pathways: purine, selenium, and sulfur metabolism. APS sulfurylase is also found in plants, where it is involved in sulfur metabolism as well [4]. In the bacterium *Desulfovibrio vulgaris* Hildenborough, APS sulfurylase is encoded by the *sat* gene. This gene is localized on the negative strand in the position 1,387,712–1,388,995. It is composed of 184 nucleotides, which are translated into 427 amino acids. The active site consists of 142 amino acids at position 209–350. The polypeptide chain contains two protein motives, a flexible turn and an HXXH motive. The HXXH motive participates in catalytic reactions. These motives are typical for proteins from the nucleotidyltransferase protein family [46].

### 3.2. Cytoplasmic APS Reduction

**Figure 2:**
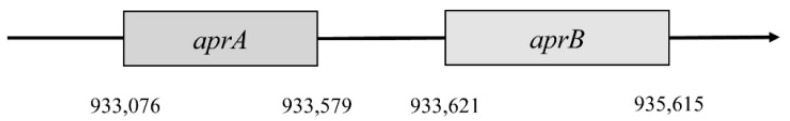
Localization of *aprA* and *aprB* genes.

This reaction is catalyzed by the enzyme APS reductase (EC 1.8.99.2), which enables the reduction of APS to sulfite or bisulfite and adenosine monophosphate (AMP) [42,43]. In the bacterium *D. vulgaris*, sulfite exists only as an intermediate [28]. This reaction is catalyzed by the enzyme APS reductase. It can be found in SRB and also in other sulfuric bacteria or in *Thiobacillus*. APS reductase is a nonheme flavoprotein that consists of two subunits: α and β [44]. In the genome of *Desufovibrio vulgaris*, Hildenborough, genes encode both subunits, and they are situated in a positive operon strand (Figure 2) [46].

The first gene encodes subunit β. It is named aprB and is situated in the 933,076 to 933,579 position. This gene consists of 504 nucleotides that are translated into 167 amino acids. Subunit β has two domains 4Fe–4S; these are encoded by 49 amino acids, forming a place for cofactors. Gene *aprA* encodes subunit α; this gene is in position 933,621–935,165. It consists of 1995 nucleotides, which are translated into 644 amino acids. This subunit reacts with flavin adenine dinucleotide (FAD) [4,46]. The gene for subunit α is almost four times larger than the *aprB* gene. Both genes must be correctly translated into proteins, and then they bind with FAD and iron. This complex is a fully functional holoenzyme that is called APS-reductase [4].

### 3.3. Cytoplasmic Sulfite Reduction

Another essential reaction of the process is sulfite reduction, resulting in the product of APS reduction [4,44]. Sulfite reduction is catalyzed by dissimilatory sulfite reductase (EC 1.8.99.1). This enzyme reduces sulfite to sulfate [44]. Sulfide reductase also plays an important role in the process of assimilatory sulfate reduction due to sulfide ion production. These sulfides are part of amino acids containing sulfur, such as methionine and cysteine. SRB may have several types of sulfide reductases that can be used for identification. These reductases are desulfoviridin, desulforubin, desulfofuscidine, and protein P_582_ [4].

**Figure 3:**
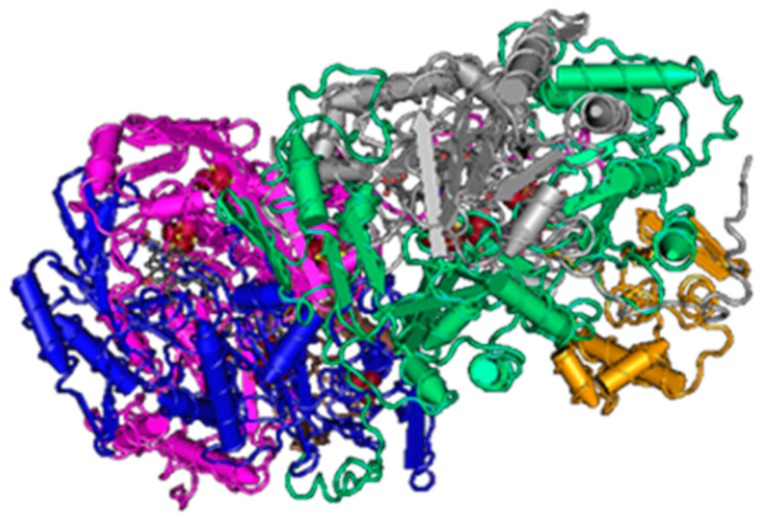
The structure of sulfite reductase (3*D* structure) [47].

Sulfite reduction is the last reaction in the process of DSR. Reactive sulfite is converted into toxic sulfide, and then it is released out of the bacterial cell [48]. This reaction is catalyzed by the enzyme sulfite reductase (Figure 3). 

Various species of SRB have different types of this enzyme (desulfoviridin, desulforubidin, desulfofuscidine, P_582_). The best-known type is desulfovidirin that can be found in the genus *Desulfovibrio*. In 2010, Barton and Hamilton discovered that this enzyme consists of 3 subunits α, β, γ. They proved the existence of the hexamer structure 2α2β2γ [44]. Every single subunit is encoded by its own gene, but they are all located in the positive strand of the chromosome. Desulforubidin is typical for genera *Desulfohalobium*, *Desulfosarcina*, *Desulfomicrobium*, *Desulfocurvus*, *Desulfobulbus*, *Desulfofustis*, and *Desulfobacter*. Desulforubidin has a very similar structure to desulfoviridin [1]. Desulfofuscidin consists of only two subunits, α and β and has been isolated from thermophilic SRB (*Thermodesulfovibrio*) [49,50,51].

In *Desulfovibrio vulgaris*, Hildenborough, subunit α is encoded by the gene *dsvA*. This gene is in the position 449,888–451,201. It consists of 1314 nucleotides that are translated into a protein with a length of 437 amino acids, and part of this protein is also the domain 4Fe–4S with a length of 231 amino acids [4]. This part belongs to the protein family Nir-Sir. Next to gene *dsvA* is gene *dvsB* that encodes subunit β. This gene is in position 451,220–452,365 and contains 1146 nucleotides that are translated into a protein with a length of 222 amino acids. The last gene called *dsvC* is responsible for the subunit γ that is located in a different part of the chromosome (position 2,883,462–2,883,779). This protein consists of 105 amino acids [46].

**Figure 4:**
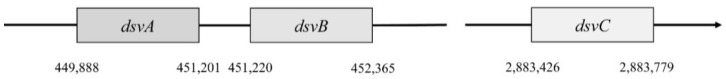
Localization of *dsvA*, *dsvB*, *dsvC* genes.

Genes *dsvA* and *dvsB* are translated as operons (Figure 4), and they form a tetramer structure from subunits α and β, and thus they comprise the center of sulfite reductase.

Their translation into proteins is coordinated together. The expression of the *dsvC* gene is separate and independent [48]. There is no more information about the expression of this gene or about how it works with other subunits [52].

### 3.4. Periplasmic Oxidation of Molecular Hydrogen

Oxidation of H_2_ occurs in the periplasm by periplasmic dehydrogenases. These hydrogenases are enzymes that catalyze reversible reactions in the presence of hydrogen. They are crucial for anaerobic respiration.

### 3.5. Transmembrane Transfer of Electrons

This process takes place in the periplasm as well. Protons are transferred to cytochrome *c*_3_ by periplasmic dehydrogenase [44].

### 3.6. Cytoplasmic Oxidation of Molecular Hydrogen

This reaction is catalyzed by cytoplasmic hydrogenases and FeS proteins.

**Figure 5:**
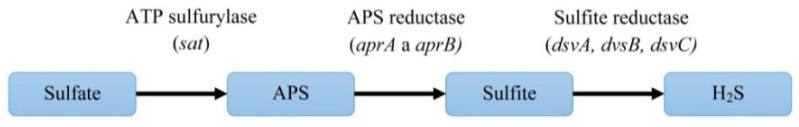
The pathway of dissimilatory sulfate reduction and the genes encoding the enzymes of this process.

The first three enzymatic reactions are important for sulfate reduction to hydrogen sulfide (Figure 5). Enzymes of mentioned reactions are connected directly with inorganic sulfate and they are typical for the DSR pathway. The next three reactions are linked to electron transport.

## 4. Hydrogen Sulfide as a Final Product of DSR and its Important Role in the Gut

Hydrogen sulfide (H_2_S) is the terminal product of SRB, secreted during the process of DSR [53,54,55]. Organic H_2_S can be produced by bacteria or by the degradation of organic material and released into the environment. The toxic effect of H_2_S was described for the first time in 1713 by B. Ramazznim. This gas is a small molecule capable of diffusing freely through membranes as well as CO and NO [56]. At normal pressure and temperature, it is a colorless, flammable gas, heavier than air, and slightly soluble in water. Its molecular weight is 34.08. H_2_S is characterized by its bad smell of rotten eggs [10].

H_2_S is toxic to most organisms, including SRB themselves. The toxicity of this compound depends on the oxidation state that may occur in three forms: H_2_S, HS^−^, S^2−^. This oxidation state is dependent on the pH of the environment [21]. The activity of SRB is influenced by the presence of heavy metals that are toxic at high concentrations, though at lower levels, they can promote SRB activity. The same applies to hydrogen sulfide. SRB are mostly able to tolerate more than 10 mM hydrogen sulfide, and DSR proceeds up to concentrations of 25 mM hydrogen sulfide in the environment, after which the bacteria begin to perish. Tolerance to H_2_S varies by species; some are more sensitive, for example, *Desulfotomaculum* (max. 4–7 mM), whereas other species are less sensitive, for example, *Desulfovibrio vulgaris* (higher than 10 mM) [57]. At high concentrations of H_2_S, the transcription of genes that encode the expression of ribosomal proteins decreases and the expression of *dsv* genes that code DSR enzymes is also strongly attenuated. On the other hand, some genes involved in proteolysis as the response to stress and iron accumulation are expressed more in response to high concentrations of H_2_S [58].

As mentioned above, increased levels of H_2_S in the gut are connected to ulcerative colitis (UC) formation. The concentration of intestinal H_2_S in healthy adult humans ranges from 0.3 to 3.4 mmol/L [59]. Hydrogen sulfide is five times more soluble in lipophilic solvents than in water and therefore penetrates through the cell membrane. It is known that H_2_S influences adenosine- 5′-triphosphate-dependent potassium channels, DNA integrity, and the activity of cytochrome oxidase and carbonic anhydrase. These effects are dependent on the hydrogen sulfide donor or inhibitors, concentrations, cells, organs or animal models used in the experiment [4,59,60].

It has been discovered that butyrate oxidation in colonocytes is destroyed by H_2_S; 70% of the energy required by the colonocytes is dependent on the butyrate formed from the fermentation of intestinal substrates by the gut microbiota. Consequently, the hypothesis of "energy deficiency" as a potential cause of UC could be suggested [61].

## 5. The Relationship of Sulfate Reduction Pathways and Inflammatory Bowel Disease (IBD)

Sulfate consumption and its conversion to hydrogen sulfide affect the pH of the intestines. The colon is an unfavorable environment for SRB due to pH lower than 5.5. By contrast, the distal part of the colon has neutral pH and is an optimal environment for SRB. The acidic pH of the intestines influences the occurrence of inflammatory bowel diseases (IBD) [4,9].

Ulcerative proctitis occurs in the most distal part of intestine and the rectum, distal colitis in the descending colon, and pancolitis in the entire colon [59,61]. The reason for the occurrence of ulcerative colitis and IBD has not been fully revealed yet. Factors, such as dietary habits and intestinal microbiome composition, influence IBD prevalence [26]. A connection between the SRB intestinal occurrence and IBD has been found. However, these bacteria are still considered an ordinary component of the digestive system of healthy people. It was calculated that the healthy population has SRB prevalence in the digestive system from 12% to 79% [11,12].

SRB can be considered only a contributing factor to ulcerative colitis development since they are not direct pathogens [12]. The main role of SRB in IBD can be explained by their production of hydrogen sulfide due to inhibition of butyrate oxidation. Without butyrate oxidation, colon cells starve since they cannot oxidase short chain fatty acids (mainly butyrate). On the other hand, apoptosis of colonic cancer cells is supported by short chain fatty acids [59]. Lower butyrate oxidation levels were observed in studies that included UC patients. The normal concentration of hydrogen sulfide in the gut ranges from 1.0 mM to 2.4 mM. These concentrations are even considered beneficial for the colonic mucosa (cell respiration is increased) [61].

Food and beverages are sources of sulfate; approximate daily intake is from 2 mmol to 9 mmol. These amounts are mainly utilized and only around 0.5 mmol is measured in daily fecal matter. The complexity of the gut microbiota can also be seen by symbiotic processes between SRB and saccharolytic bacteria. Saccharolytic bacteria disengage bound sulfate [11].

SRB influence the prevalence of IBD, but they are certainly not the only factor. The production of hydrogen sulfide affects the colon environment, but excessive amounts of hydrogen sulfide are also toxic for SRB [5].

As already mentioned, hydrogen sulfide is the final product in the metabolism of SRB in the dissimilatory sulfate reduction process. SRB represent the main hazardous issues considering the occurrence of SRB in the gut microbiota. Thus, the pathway switch from DSR to ASR can be seen as the opportunity to decrease hydrogen sulfide formation by intestinal SRB or at least its production in lower quantities. 

## 6. Assimilatory Sulfate Reduction

The metabolic pathway of ASR occurs entirely in plants and microorganisms. These organisms can utilize sulfur in a reduced state. In contrast to other elements, such as carbon, nitrate and sulfur go through complex transformations in living organisms (including humans, intestinal bacteria, and plants) [22]. Sulfur as opposed to carbon or nitrate can be utilized in the highest oxidation state (sulfate). ASR is necessary for the formation of sulfur-containing amino acids. Many organisms reduce sulfate to thiol that is a part of coenzymes or amino acids, such as cysteine or methionine. These serve as a structural block for proteins and polypeptides. An essential step in this metabolic pathway is the same as in DSR, that is, sulfate activation in reaction with ATP. Another reaction is the formation of 3′-phosphoadenosine-5′-phosphosulfate (PAPS). This step is special only for assimilatory sulfate reduction (see Figure 1). There are two possible pathways for the reduction [22,47].

The first one occurs in *Cyanobacteria* and plants. The pathway is the typical formation of APS that is transformed by the enzyme APS sulfotransferase to organic thiosulfate, followed by reduction by thiosulfate reductase to glutathione. The glutathione reacts with *O*-acetyl serine and catalyzed by *O*-acetyl serine-(thiol)-lyase. The final product of this pathway is l-cysteine.

The second pathway occurs only in bacteria and yeast. This pathway runs through intermediate PAPS that is reduced to sulfite that is catalyzed by sulfite reductase to sulfide. Then, sulfide is transformed to l-cysteine by *O*-acetyl serine-(thiol)-lyase [22].

l-cysteine synthesis from inorganic sulfate is the dominant mechanism for sulfur incorporation into organic compounds. In case that inorganic sulfate is unavailable, there are mechanisms for l-cysteine formation from organic products. Sulfate is the most abundant source of sulfur in the biosphere. In plants and many microorganisms, including intestinal bacteria such as *Salmonella enterica* serovar *typhimurium* and *E. coli*, sulfate plays a key role in sulfur metabolism. The mechanism of sulfur fixation is very similar to the fixation of ammonium to glutamine or glutamate [61]. ASR plays a role in the production of amino acids and is regulated by cysteine regulon. This pathway differs in many steps from DSR, where sulfate is used as the terminal acceptor and for the production of large amounts of hydrogen sulfide. ASR can be divided into several steps.

Sulfate transfer—in this first step, inorganic sulfate must be transported through the cytoplasmic membrane. It is catalyzed by the enzyme sulfate thiosulfate permease (EC 3.6.3.25) [61].

1. Activation of sulfate—in the beginning, this reaction is the same as in the DSR pathway, but adenosin-5-phofphosulfate is phosphorylated by APS kinase and the product is 3-phosphoadenosine-5-phosphosulfate (PAPS) [62].
2. Reduction of 3-phosphoadenosine-5-phosphosulfate—the reaction is catalyzed by PAPS sulfotransferase, and PAPS is reduced to sulfite [22].
3. Sulfite reduction-bacteria utilize various types of sulfite reductase according to the amount of oxygen in the environment. For example, in *Salmonella typhimurium* or in *E. coli*, there is special NADPH-sulfite reductase for aerobic conditions that consists of two monomers, flavoprotein and hemoprotein, and both are crucial for the function. Sir-FP accepts electrons from NADPH and transfers them to Sir-HP that reduces sulfite to sulfide [63].
4. Cysteine synthesis—l-cysteine is formed from *O*-acetyl-l-serine. *O*-acetyl-l-serine reacts with sulfide and creates l-cysteine. This reaction is catalyzed by *O*-acetylserine-(thiol)-lyase. These reactions take place in a multifunctional structure called cysteine synthetase [64,65].

ASR is more complicated than the dissimilatory pathway. The reason is that facultative anaerobic microorganisms must be equipped with enzymes for anaerobic and aerobic conditions. DSR needs only three enzymes because SRB are strictly anaerobic organisms. In the assimilatory pathway, seven different enzymes are necessary for the right function.

### 6.1. Sulfate Transport to Bacterial Cells

As mentioned in *E. coli*, K-12 substr. MG1655 sulfate transportation is catalyzed by the enzyme sulfate permease. Subunits for the enzyme are encoded by genes *cysP*, *cysU*, *cysW*, *cysA,* and *cbp*. There is another gene, *cysZ*, described in the literature; however, it is located out of the cysteine regulon and its deletion has no impact on the function. All genes are located on the negative chromosome strand. The first is gene *cysP*, located in position 2,542,512–2,543,528. It contains 1017 nucleotides that encode 338 amino acids. These subunits form the ABC periplasmatic thiosulfate–sulfate transporter. The second one is *cysU* that contains 834 nucleotides, forming a protein with a length of 277 amino acids. This protein binds to the inner cytoplasmic membrane. The gene *cysU* is located in the position 2,541,679–2,542,512. The third gene *cysW* is located in position 2,540,840-2,541,679. It forms a protein consisting of 291 amino acids that are encoded by 876 nucleotides. *CysA* is the last gene creating part of sulfate permease from the cysteine regulon. It is situated in position 2,539,717–2,540,814. This protein is encoded by 1089 nucleotides, translating into 365 amino acids. The gene *sbp* encodes another subunit of the ABC thiosulfate–sulfate transporter; however, it is situated in position 4,108,834–4,109,823 out of the cysteine regulon. It consists of 990 nucleotides and forms a protein with a length of 329 amino acids [66].

**Figure 6:**
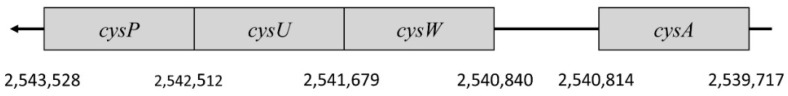
Cluster cysPUWA.

The whole complex of genes creates cluster cysPUWA (Figure 6), meaning that genes are located side by side. This cluster is described in *E. coli* and *S. typhimurium*. Genes *cysU* and *cysW* form homologous peptides that probably create a channel for sulfate and thiosulfate transport [67].

### 6.2. Sulfate Activation

**Figure 7:**
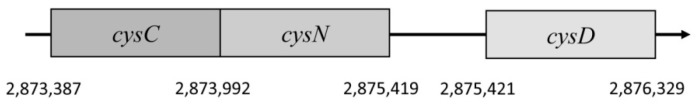
Cluster *cysCND*.

Sulfate must be activated after transportation into the bacterial cell. This reaction is the same as in the dissimilatory pathway mentioned above. APS is phosphorylated by the enzyme APS kinase. This enzyme occurs, for example, in *E. coli* K-12 substr. MG1655. It is encoded by three genes, *cysC*, *cysN*, and *cysD*. These are situated on the negative chromosome strand as well. They are part of the cysteine regulon and create cluster *cysCND* (Figure 7). The gene *cysC* encodes an enzyme APS kinase; this gene is located in position 2,873,387–2,873,992. It has 606 nucleotides and they are translated into 201 amino acids. In the case that the enzyme is phosphorylated, it forms a dimer, compared with a tetramer in the free form [68].

The next gene is located in position 2,873,992–2,875,419 and it is called *cysN*, consisting of 1428 nucleotides. This gene encodes a subunit which has a catalytic function in the enzyme. The last subunit is encoded by gene *cysD* and it has 909 nucleotides. They form a protein consisting of 302 amino acids. In the *E. coli* genome, gene *cysD* is situated in position 2,875,421–2,876,329. These two subunits are not homological, and they create an important part of the enzyme APS sulfurylase.

### 6.3. Reduction of 3-Phosphoadenosine-5-Phosphosulfate

**Figure 8:**
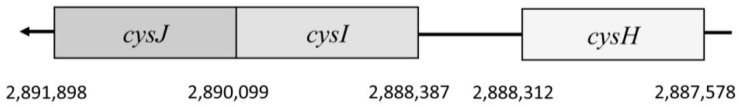
Cluster *CysJIH*.

Many different organisms can synthetize PAPS as a donor for the formation of ester sulfate. On the other hand, the reduction of PAPS occurs only in microorganisms and plants [69]. This reaction is catalyzed by the enzyme phosphoadenosine phosphosulfate reductase; its dimer consists of two completely homological subunits that are encoded by one gene called *cysH*. In the genome of *E. coli* K-12 substr., MG1655 is placed in the position 2,887,578–2,888,312. It is 735 nucleotides long and forms a protein consisting of 244 amino acids. This gene is a part of the *cysJIH* operon (Figure 8), comprising genes for sulfite reductase subunits [70].

### 6.4. Sulfite Reduction

Sulfite reductase in *E. coli* K-12 substrain MG1655 is an enzyme formed by two protein monomers, flavoprotein and hemoprotein. The two monomers are linked together to form a functional holoenzyme. For proper enzyme function, a cofactor is required by sulfur. The genes encoding the flavoprotein and hemoprotein subunits are part of the cysJIH operon. The sirohem cofactor gene is located outside the cysteine regulon. A holoenzyme structure was prepared in vitro, indicating that the enzyme has an α8β8 structure [71].

The first transcribed gene is *cysJ*. This gene encodes a flavoprotein monomer (Sir FP). It is 1800 nucleotides long and forms a 599-amino acid protein. It lies on the chromosome in the region of 2,890,099–2,891,898 [66].

This is followed by the *cysI* gene, which encodes a hemoprotein monomer (Sir-HP). This gene is located on the chromosome at 2,888,387–2,890,099. It consists of 1713 nucleotides, which are translated into the final product that is 570 amino acids long. Sir-HP contains an Fe_4_S_4_ cluster and a sulfur cofactor. This cofactor is typical for sulfite and nitrite reductase [63].

The *cysG* gene for sire synthesis lies outside the cysteine regulon. It lies on the positive strand of the chromosome at 3,497,828–3,499,201. It is 1374 nucleotides in length and forms a 457-amino acid protein. This enzyme catalyzes the formation of the sulfuric moiety of sulfite reductase. Its synthesis is a limiting factor in the expression of NADH sulfite reductase from a plasmid [72].

## 7. Cysteine Biosynthesis

Cysteine synthesis is catalyzed by a multifunctional complex called cysteine synthase. It consists of serine transacetylase and *O*-acetylserine-(thiol)-lyase that occurs in two isoenzymes, designated A and B. This complex catalyzes the acetylation of l-serine to *O*-acetyl-l-serine that is the direct precursor to l-cysteine. *O*-acetyl-l-serine reacts with sulfide to form l-cysteine with *O*-acetylserine-(thiol)-lyase. Cysteine synthase has a mass of about 309 KDa [48]. Each unit consists of four *O*-acetylserine-(thiol)-lyase subunits and six serine transacetylase subunits [73,74]. Serine transacetylase is encoded in the *E. coli* genome K-12 substr. MG1655 on the negative strand by the *cysE* gene in the region 3,781,741–3,782,562. This gene lies outside the cysteine regulon; it is 822 nucleotides in length, and the resulting product has a length of 273 amino acids. Serine transacetylase occurs normally in a cysteine synthase complex in a bacterial cell and is much less abundant than *O*-acetylserine-(thiol)-lyase [65].

Most important for the formation of cysteine are *O*-acetylserine-(thiol)-lyase A and B, each encoded by its own gene. The *cysK* gene for *O*-acetylserine-(thiol)-lyase A is found on the positive strand at 2,532,409–2,533,380, consisting of 972 nucleotides that translate into a protein 323 amino acids long. The *cysM* gene that encodes *O*-acetylserine-(thiol)-lyase B lies on the negative strand of the chromosome at 2,538,672–2,593,583. It consists of 912 nucleotides that produce a 303-amino acid-long protein [66].

Both of these enzymes form dimers and can exist in the free form and bound in the cysteine synthase complex. By comparing the amino acid sequence of *E. coli*, these isoenzymes are 43% identical. The mutation probably does not significantly affect growth under aerobic conditions, but *O*-acetylserine-(thiol)-lyase A has 10 times greater activity under aerobic conditions, whereas *O*-acetylserine-(thiol)-lyase B is important for growth under anaerobic conditions and for growth in thiosulfate [74].

## 8. Cysteine as a Final Product of Assimilatory Sulfate Reduction

Cysteine is the terminal product of ASR in a bacterial cell. Together with methionine, it is one of the proteinogenic amino acids containing sulfur. In cysteine, sulfur occurs as a reactive thiol group. Cysteine is soluble in water and forms hydrogen bridges. Cysteine has uncharged polar amino acids. Due to high reactivity in an alkaline environment, enzymes in active sites are often used. The reaction forms disulfide bridges between cysteines and thereby links polypeptides [75].

The role of cysteine is mainly in the formation of coordinating the binding of metal ligands in structures such as zinc fingers or in cytochrome P_450_, NiFe hydrogenases, and many others [76]. In addition to being part of proteins, cysteine is also a precursor for a large number of essential biomolecules such as vitamins and/or antioxidants, such as glutathione that is responsible for maintaining redox homeostasis [77]. All mentioned molecules, where sulfur forms a functional group, are derived from cysteine. The biosynthesis of cysteine proceeds in two successive steps. The first is the formation of *O*-acetylserine. This reaction is catalyzed by the enzyme serine acetyltransferase. The following reaction is the formation of cysteine itself from sulfide and *O*-acetylserine with the participation of *O*-acetylserine-(thiol)-lyase. Together, these enzymes form a heteromeric cysteine synthase complex that was described in bacteria [78].

## 9. Summary: Comparison of Dissimilatory and Assimilatory Sulfate Reduction and the Prospects for Further Research

DSR and ASR are two metabolic pathways that differently metabolize sulfate in a bacterial cell. The first pathway uses sulfate as a terminal acceptor and is indispensable for cells because it generates energy for ATP production; the terminal product is toxic hydrogen sulfide [41,79,80,81]. This pathway is preserved in a few bacteria, whereas ASR uses sulfate to form the amino acid cysteine that is indispensable for living organisms. This metabolic pathway occurs not only in prokaryotic organisms, but also in plant eukaryotic cells. However, this metabolism does not exist in animal eukaryotic cells and cysteine can only be obtained from food [22].

In *D. vulgaris* and *E. coli*, significant differences can be observed between the two metabolisms, both at the enzymatic and gene levels. While the DSR in *D. vulgaris* comprises three enzymes, adenylyl transferase sulfate, APS reductase, and sulfite reductase, the ASR in *E. coli* contains up to seven enzymes. The differences are not only in the number of enzymes, but also in the structure of the same enzymes, for example, in adenylyl transferase sulfate, in which *E. coli* is composed of non-homologous subunits. On the other hand, some enzymes have a very similar structure to sulfite reductase that is almost identical in both bacteria. The most significant difference is that the dissimilatory pathway results in sulfite reductase and hydrogen sulfide production, while in ASR, hydrogen sulfide is further metabolized to cysteine by the cysteine synthase enzyme. 

Other significant differences can be found at the gene level, where the most significant difference is that the ASR enzyme genes are all located in the so-called cysteine regulon [81]. It contains most of the important genes for proper function in a certain region of the chromosome. *D. vulgaris* genes of DSR are distributed differently over the genome.

Though the two metabolic pathways are different in many parts, there is the possibility of converting the dissimilatory pathway, the terminal product of which is toxic hydrogen sulfide, to assimilatory sulfate reduction, where the product is harmless cysteine. This change would theoretically be possible through horizontal gene transfer.

Certainly, there is enough literature information about SRB and their importance in inflammatory bowel diseases including ulcerative colitis in animals and humans [82]. However, the solution to this problem has not yet been described. The treatment of these diseases usually involves the administration of antibiotics and other antimicrobial agents that inhibit other types of intestinal bacteria and cause dysbiosis [55]. One possibility is to change the dissimilatory pathway of SRB to the assimilatory pathway, where the product is harmless cysteine. This change can be accomplished by horizontal gene transfer with a plasmid.

Boronat et al. isolated plasmid pAB65 from the *E. coli* genome that encodes two polypeptides with a molecular weight (Mr) of 34,000. One of these two polypeptides was further identified as an *O*-acetylserine-(thiol)-lyase subunit. The amino acid sequence and molecular weight of the enzyme subunits from the plasmid were determined, and it was shown that this enzyme coincides with the *cysK* gene product of *S. typhimurum* [83].

## 10. Conclusions

Intestinal bacteria are an integral part of the human intestinal tract and have a significant effect on human health. They are involved in maintaining homeostasis in the body and play a key role in providing nutrients and digesting food. In the human intestine, there are up to 10^14^ bacterial cells and from these, up to 2172 different species have been identified. Bacterial colonization depends on many factors. In different parts of the digestive system, there are different types of bacteria, typical for that part, depending on the available substrate. In the small intestine, bacteria can found in sugars that are not absorbed. In the large intestine, it is rather anaerobic bacteria that gain energy by the fermentation or sulfate by reduction. Fatty acids and gases are the products of the metabolism of these bacteria. Fatty acids are essential for the proper functioning of enterocytes, since they are used as a source of energy. Gases released into the intestine (such as H_2_S) may be used by other bacteria as a source of electrons, though some (H_2_S) may be toxic.

Many different substances and compounds are present in the diet, one of which is sulfate, which is present in industrially processed foods, but also in vegetables and some drinks. Sulfate is poorly absorbable in the intestine and is used by SRB as an electron acceptor in dissimilatory sulfate reduction, the final product of which is H_2_S. Sulfate is not only used by SRB, but also other types of bacteria involved in the sulfur cycle in nature. These bacteria do not use sulfur in the DSR, but in assimilatory sulfate reduction.

DSR is a metabolic pathway that is used to gain energy. In this process, organic substances (lactate, acetate, butyrate, etc.) are oxidized and sulfate is reduced to H_2_S. Several enzymes are involved in this process. Important for reducing sulfate to H_2_S are APS sulfurylase encoded by the *sat* gene, APS-reductase encoded by the *aprA* and *aprB* genes, and the sulfite reductase enzyme that is encoded by the three genes, *dsvA*, *dvsB*, and *dsvC*. *D. vulgaris* Hildenborough has been described with respect to genome localization and length.

In assimilatory sulfate reduction, sulfate is used to form cysteine. This is essential for organisms because it serves as a building block for proteins and various enzymes. This metabolic pathway is different from the previous one because it contains more enzymes that have a more complex structure. ASR includes the enzyme sulfate permease that is encoded by the *cysPUWA* operon and the gene outside the sbp operon, ATP sulfurylase encoded by the *cysDNC* cluster, phosphoadenine phosphosulfate reductase encoded by the *cysH* gene that is the part of the *cysJIH* gene, and *O*-acetylserine-(thiol)-lyase A and B encoded by the *cysK* and *cysM* genes. Their location and the length in the genome of *E. coli* K-12 substr MG1655 were described.

Due to the toxicity of H_2_S, there is an opportunity to change DSR to assimilatory sulfate by means of molecular biology methods and thereby limit its production. The change of DSR to assimilation involves the isolation and amplification of the gene *O*-acetylserine-(thiol)-lyase B and its introduction into the vector and transfer by plasmid into SRB bacterial cells or utilization of the already prepared plasmid pAB65. Therefore, further research should focus on realizing the possibility of prolonging the metabolic pathway from hydrogen sulfide to cysteine in bacteria using DSR. The above findings suggest that this realization is possible.
